# Field-Based and Lab-Based Assisted Jumping: Unveiling the Testing and Training Implications

**DOI:** 10.3389/fphys.2018.01284

**Published:** 2018-09-12

**Authors:** James J. Tufano, Jan Malecek, Michal Steffl, Petr Stastny, Vladimir Hojka, Tomas Vetrovsky

**Affiliations:** ^1^Department of Physiology and Biochemistry, Faculty of Physical Education and Sport, Charles University, Prague, Czechia; ^2^Department of Sport Games, Faculty of Physical Education and Sport, Charles University, Prague, Czechia; ^3^Department of Track and Field, Faculty of Physical Education and Sport, Charles University, Prague, Czechia

**Keywords:** plyometric, assist, jump, impact, power, perceived exertion

## Abstract

**Purpose:** Assisted jumping can supplement resistance training and traditional plyometric training to increase vertical jump performance. However, as coaches may choose to make field-based decisions based on lab-based research, this study determined whether a field-based assisted jumping set-up results in different ground contact times (CT), take off forces (TOF), flight times (FT), and impact forces (IF) compared to a lab-based set-up.

**Methods:** Eighteen active males (24.8 ± 3.0 yr; 178.8 ± 7.8 cm; 77.8 ± 7.8 kg) performed two sessions of assisted jumping: one with each hand holding a commercially available resistance band (1m) that was attached to a pull-up bar (_FIELD_), and the other with assistance from a custom-built system of ropes, pulleys, and long (3 m) elastic bands (_LAB_). With each set-up, subjects performed five sets of five countermovement jumps on a force plate. Each set was performed with either bodyweight (BW), 90, 80, 70, or 60% of BW, which was achieved by either grabbing higher or lower on the bands during _FIELD_, or by being pulled upward via a full-body harness during _LAB_. The order of each visit was counter-balanced, and the order of jumps within each visit was quasi-randomized. Data from the 90, 80, 70, and 60% trials for each set-up were then expressed relative to the data of BW jumps, and these relative values were then used for analysis.

**Results:** CT_FIELD_ was less than CT_LAB_ at 80, 70, and 60%. FT_FIELD_ was greater than FT_LAB_ at 90 and 80%, but FT_LAB_ became greater at 60%. TOF and IF remained unchanged during _LAB_, but TOF_FIELD_ was consistently less than TOF during BW, with IF_FIELD_ generally being greater than IF_LAB_.

**Conclusion:** If the purpose of assisted jumping is to spend less time on the ground without decreasing force, systems with finite adjustments and longer bands like _LAB_ should be used. However, shorter bands similar to _FIELD_ may also be used; but due to the larger variability of assistance throughout the range of motion, such systems may alter the neuromuscular characteristics of the jump in other ways that should be investigated in future research.

## Introduction

As athletes and coaches continually devise novel training strategies to increase power output and performance, researchers and practitioners often work together to determine the effectiveness of these strategies. For example, it is common to see different resistance training configurations of sets, repetitions, loads, and rest periods both in practice and in the scientific literature (Tufano et al., 2016; Walker et al., 2016), but the large majority of these strategies are similar in that they overload an athlete, technically focusing on the force aspect of power output. Since previous researchers have recommended that athletes aiming to increase their power production at bodyweight tasks such as sprinting and jumping should train with loads that span across the entire force-velocity spectrum (Cormie et al., 2007; Cormie et al., 2011), bodyweight exercises are often used in addition to loaded exercises. Some coaches even go so far as to utilize assisted training to “reduce" bodyweight, expanding the force-velocity spectrum to include exercises at what can be considered as supra-maximal velocities (Sheppard et al., 2011; Tran et al., 2012; Cazas et al., 2013; Tufano and Amonette, 2018).

Since previous research has shown that performance is improved at or near the velocities utilized during training (Kanehisa and Miyashita, 1983; Behm and Sale, 1993; Brown and Whitehurst, 2003; Murray et al., 2007), the underlying theory of assisted training is based on the principle of specificity (Markovic et al., 2011; Tran et al., 2012; Tufano and Amonette, 2018). As such, it could be hypothesized that training with supra-maximal velocities could result in neuromuscular adaptations that increase one’s maximal movement velocity, which would likely be showcased during bodyweight movements such a running and jumping. Compared to resisted training which overloads the force aspect of power output and subsequently decreases movement velocity, athletes tend to neglect over-speed training that may exploit an untapped potential for maximizing the velocity aspect of power output, especially in athletes who already have a high level of strength but lack the ability to produce higher movement velocities at low loads (Argus et al., 2011). For example, assisted jumping can be used to acutely decrease an athlete’s bodyweight with the aim of resulting in an over-speed stimulus by moving faster (Markovic and Jaric, 2007; Argus et al., 2011), spending less time on the ground (Makaruk et al., 2014), and jumping higher (Tran et al., 2011), all of which would be desirable training adaptations for athletes who must quickly propel their own bodyweight during competition (e.g., jumping and sprinting). Additionally, as assisted jumping results in a greater jump height than bodyweight jumping, flight time increases which increases the time that gravity accelerates an athlete to the ground, possibly increasing impact forces that could result in eccentric strength adaptations, changes in jump kinematics, and increased jumping performance (Walsh et al., 2004; Papadopoulos et al., 2014; Matic et al., 2015). Although these ideas are supported by research (Tran et al., 2011), and coaches and practitioners often use resistance bands to provide assistance and “unload” an athlete during training (Kilgallon and Beard, 2010; Darmiento et al., 2012), these field-based set-ups with shorter resistance bands likely have different elastic properties than the custom-built assisted systems with finite adjustments and longer elastic bands that researchers often use (Cavagna et al., 1972; Markovic and Jaric, 2007; Markovic et al., 2013), which may ultimately alter the effectiveness of assisted training strategies.

Since the purpose of assisted jumping is to alter the kinetics of jumping and since coaches may choose to base their decisions in the field on findings made in the laboratory, it would be sensible to determine whether field-based assisted methods result in different kinetics than the lab-based methods that are present in the literature: a comparison that is needed (Tran et al., 2012), but has not yet been conducted. As most lab-based systems are constructed using a system of pulleys and long elastic bands that are designed to provide a constant and precise assistance level, it could be possible that the shorter elastic bands used in the field may not mimic the optimal assistance levels that have been previously observed in the laboratory. Therefore, the purpose of this study was to determine whether a field-based system using commercially available resistance bands results in different ground contact and flight times, take-off forces and impact-forces, and perceptual responses compared to a custom-built lab-based assisted jumping system using the same initial assistance levels.

## Materials and Methods

### Subjects

Eighteen active males participated in this study (24.8 ± 3.0 yr; 178.8 ± 7.8 cm; 77.8 ± 7.8 kg). To be included in the study, subjects must have been able to perform countermovement jumps, must have participated in at least recreational-level sports training that periodically involved jumping for the past 3 years, and must not have had any recent musculoskeletal injuries. All procedures were carried out in accordance with the Declaration of Helsinki and were approved by the Charles University Faculty of Physical Education and Sport Ethics Committee (028/2018). All participants gave written informed consent prior to participating.

### Design

Testing occurred over two sessions that were counterbalanced and performed 48–96 h apart: a lab-based testing session (_LAB_) and a field-based testing session (_FIELD_). During each session, subjects completed a single set of five consecutive maximal-effort countermovement jumps with bodyweight (BW), 90, 80, 70, and 60% of BW (i.e., five sets of five jumps, each set with a different BW). To avoid an order effect or any potentiation effects of the previous set, each set was performed in a quasi-randomized order with 3 min of rest between sets. All jumps were performed on side-by-side synchronized force plates, from which ground contact time (CT), flight time (FT), peak take-off force (TOF), and peak impact force (IF) were assessed.

### Methodology

**FIGURE 1:**
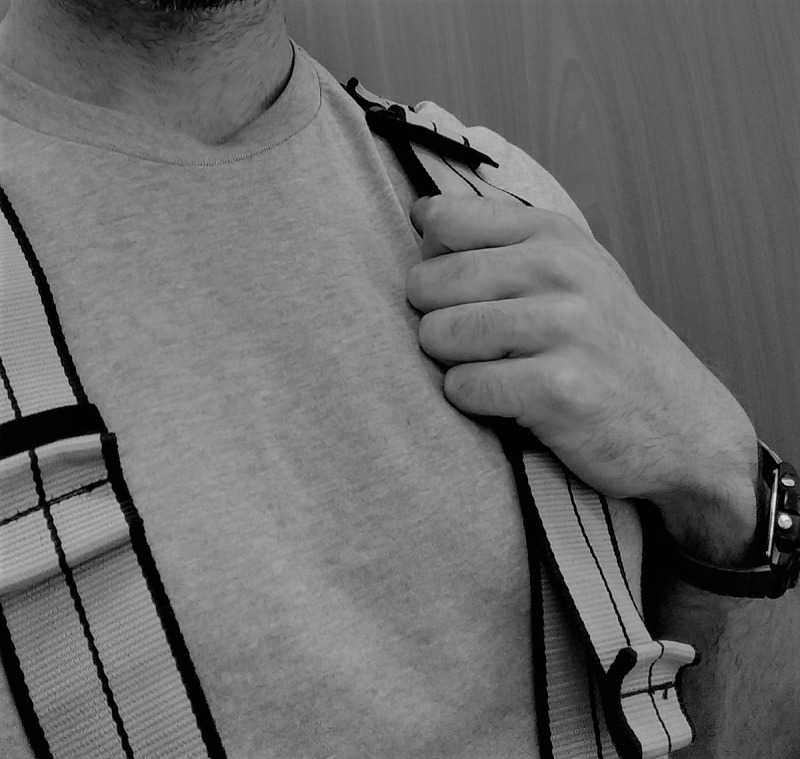
Position of the hands on the harness during all jump trials.

As the order of the sessions was counterbalanced, the first session included body mass and height measurements. Next, a dynamic warm-up was completed that consisted of various lower limb exercises (i.e., bodyweight squats, lunges, walking quadricep and hamstring stretches, leg swings, lateral lunges, high-knee running, and butt-kick running). This warm-up was identical for both sessions, and then, still for both sessions, subjects put on a full-body harness and their elbows were flexed so that the hands were able to grab the pectoral region of the harness, as this position was needed to secure the resistance bands during _FIELD_, but was also maintained during _LAB_ for the sake of testing consistency (**Figure 1**). While in this position, subjects then performed three sets of five bodyweight countermovement jumps with progressively increasing effort (75, 90, and 100% perceived maximal effort) with approximately 2 min of rest between sets. Subjects were then familiarized with the assisted jumping set-up for that day by performing five jumps with 80% BW at 75% maximal effort, as 80% BW was considered to be a moderate assistance level. The assistance was then removed, subjects rested 2–3 min, and then performed another set of five jumps with 80% BW with 100% maximal effort to complete their familiarization. In the rare event that a subject still was not comfortable with the jumping set-up or if the researchers noted inconsistent jumping and landing locations on the force plate, another rest period was given, the researcher provided further instructions, and the subject performed another set of maximal effort familiarization jumps. Following 3 min of rest, the experimental sessions began with the first of the assigned loads.

For each of the quasi-randomized BW loads (BW, 90, 80, 70, and 60% BW), subjects performed five consecutive maximal effort counter-movement jumps on the force plates. Throughout each set, subjects were verbally encouraged to “explode off the plate,” and “jump as high” as they could while holding the harness at the pectoral region. Subjects were instructed to look down at the front of the force plates during all jumps, as this cue was found to result in the most consistent landing locations during pilot testing during both the _LAB_ and _FIELD_ conditions. After the first set was completed, the assistance was removed, and subjects had 3 min of rest where they casually walked around the room at normal bodyweight. After 2.5 min, the subject stood on the force plates and the next assistance level was applied, ready to perform the next jump when the 3 min rest period concluded. This procedure was repeated until all five sets of five jumps were completed. Immediately after each set, in both sessions, the subject’s rating of perceived exertion (RPE) was recorded using a 0–10 OMNI scale.

#### Lab-Based Session

**FIGURE 2:**
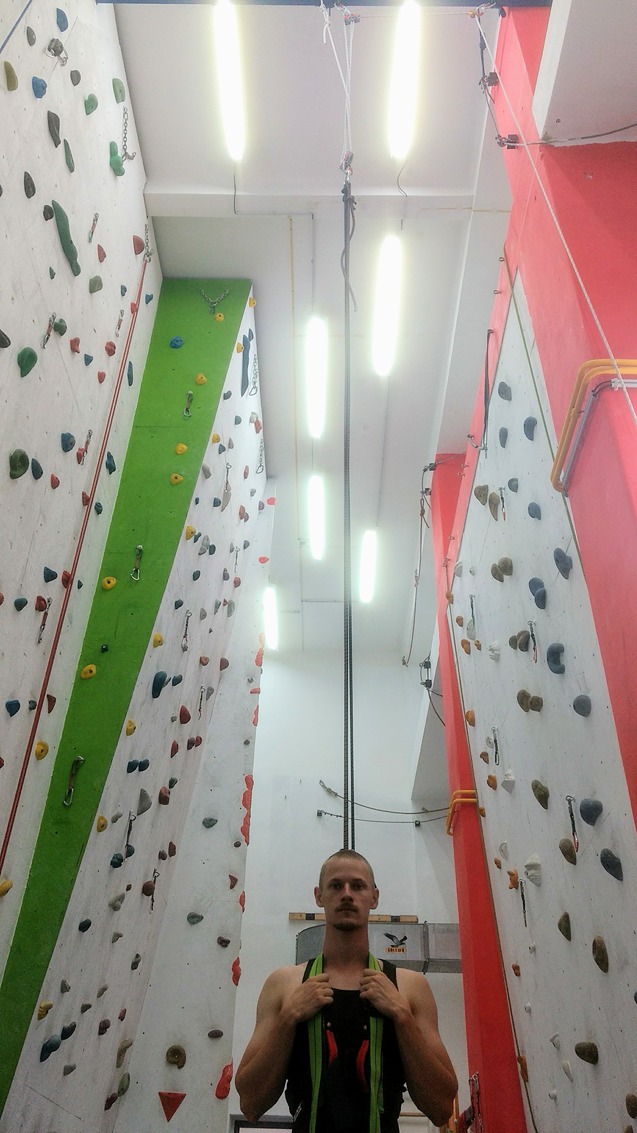
Upright position during the laboratory-based session where a system of ropes, pulleys, and elastic cords were pulled to achieve the desired amount of assistance in the standing position.

The assistance set-up in this session was constructed to mimic previous lab-based methods (Markovic and Jaric, 2007). A group of elastic bands connected the subjects to the ceiling via a system of ropes and pulleys that could be adjusted to stretch the bands and accurately provide the desired level of assistance in the standing position (**Figure 2**). To ensure that the level of assistance was as constant as possible throughout the support phase of each jump, the resting band length (3 m) was maximized according to the constraints of the laboratory’s ceiling, and the number of bands used was adjusted depending on each subject’s body mass so that the difference of the level of assistance between the standing position and full squat position was less than 10%. For subjects up to 73 kg, a group of bands was used with an elastic modulus of 352 N⋅m^-2^ and a stiffness of 343 N⋅m^-1^. For subjects 74–90 kg, the elastic modulus was 378 N⋅m^-2^ and the stiffness was 369 N⋅m^-1^. For subjects over 90 kg, the elastic modulus was 380 N⋅m^-2^ and the stiffness was 371 N⋅m^-1^. The properties of the bands were determined by measuring the change in length across a variety of static loads ranging from 2.5 to 50 kg, as the minimum assistance level needed during the upright standing position during testing was 6 kg (62 kg subject at 90% BW) and the maximum assistance level was 37.6 kg (94 kg subject at 60% BW).

#### Field-Based Session

**FIGURE 3:**
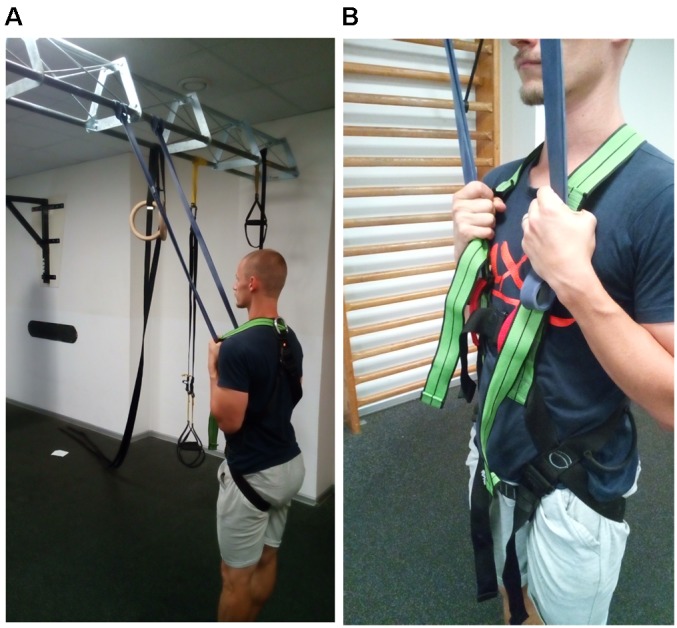
**(A)** Upright position during the field-based session where subjects held on to two resistance bands that were attached to a transverse bar in the gym. **(B)** During the field-bases session where subjects grabbed higher or lower on the resistance bands, which were then secured against the body by holding the bands between the hands and the harness.

The assistance in this session was meant to mimic what athletes likely use in practice (Kilgallon and Beard, 2010; Darmiento et al., 2012; Wilson and Kritz, 2014). Therefore, two commercially available resistance bands (DOMYOS elastic training band, ‘purple,’ 50 kg resistance, 1 m resting length) were attached to a horizontal traverse bar in the gym (2.44 m high) that would typically be used for pull-ups, suspension system attachments, and so on. The two bands were wrapped around the bar at shoulder-width and subjects grabbed the bands with their bare hands (**Figure 3A**). To minimize slippage and result in a consistent “attachment point” to the body, the resistance bands were pulled downward and manually squeezed between the hands and the body-harness, with the fingers wrapping in front of the bands and the thumbs behind the harness (**Figure 3B**). Subject weight was measured in this position and the hands were moved higher or lower on the bands to achieve the desired level of assistance in the standing position. By design, this assistance system displayed different elastic properties than the lab-based system. The properties of the _FIELD_ bands were also determined by measuring the change in length across a variety of static loads ranging from 2.5 to 50 kg, as the minimum assistance level needed during the upright standing position during testing was 6 kg (62 kg subject at 90% BW) and the maximum assistance level was 37.6 kg (94 kg subject at 60% BW). For each band, the elastic modulus was 60 N⋅m^-2^ and stiffness was 154 N⋅m^-1^ (two bands were used during all jumps in _FIELD_).

#### Force Plate Data Acquisition

All jumps were performed on two side-by-side piezoelectric force plates (Kistler 9286BA, Kistler Instruments Inc., Winterthur, Switzerland) and ground reaction forces were sampled at 2000 Hz. Signals from the force plates were amplified and recorded on a computer using a 16-bit A/D board and BioWare V5.3.2.9 software. Vertical ground reaction force data were used to identify contact time and flight time with a threshold of 20N. To determine the effects of repeated countermovement jumps during each session, the first and last jump of each set were disregarded and the mean of the middle three repetitions were used for analysis. In a custom MatLab program (1.8.0.121, MathWorks, Natic, MA, United States), CT, FT, TOF, and IF were calculated.

### Statistical Analyses

To account for possible day-to-day variations in jump height, the CT, FT, TOF, and IF of the assisted jumps were compared to the BW jumps of the same session, meaning that all data are reported relative to the values of the BW jumps on the same day. Means and standard deviations were calculated for all variables. Individual two × five (set-up × assistance level) repeated measures analysis of variance (ANOVA) were used to compare means for all variables. In the event of significant main effects and interactions, a Holm’s Sequential Bonferroni follow-up test was performed to control for Type I error and assess pair wise comparisons. Effect sizes were calculated using Cohen’s *d* and can be interpreted as small, *d* = 0.2; moderate, *d* = 0.5; and large, *d* = 0.8. To avoid an exasperating number of effect sizes, only moderate and large values are reported and discussed. Significance was set at *p* ≤ 0.05 for all tests. All statistical analyses were performed using SPSS version 22.0 (IBM, Armonk, NY, United States).

## Results

**FIGURE 4:**
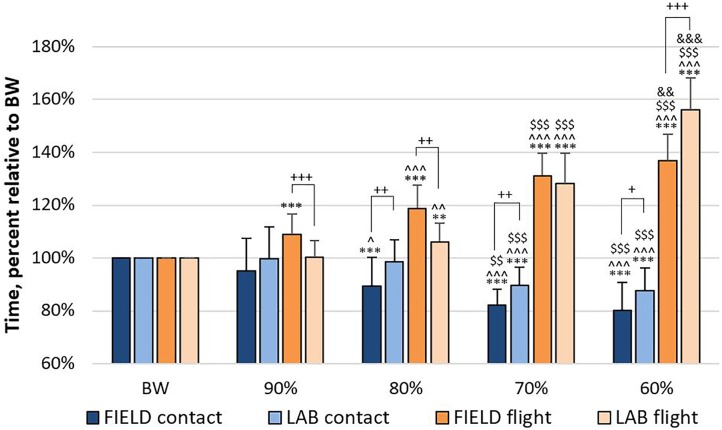
Data are expressed as mean ± *SD*. Ground contact and flight time during lab-based (LAB) and field-based (FIELD) assisted countermovement jumping at 90, 80, 70, and 60% of bodyweight (BW), expressed relative to values during BW jumps. Significantly different than BW ^∗∗∗^*p* ≤ 0.001, ^∗∗^*p* ≤ 0.01; than 90% ^∧∧∧^*p* ≤ 0.001, ^∧∧^*p* ≤ 0.01, ^∧^*p* ≤ 0.05; than 80% ^$$$^*p* ≤ 0.001, ^$$^*p* ≤ 0.01; than 70% ^&&&^*p* ≤ 0.001, ^&&^*p* ≤ 0.01. Significantly different than the other set-up at the same bodyweight ^+++^*p* ≤ 0.001, ^++^*p* ≤ 0.01, ^+^*p* ≤ 0.05.

**FIGURE 5:**
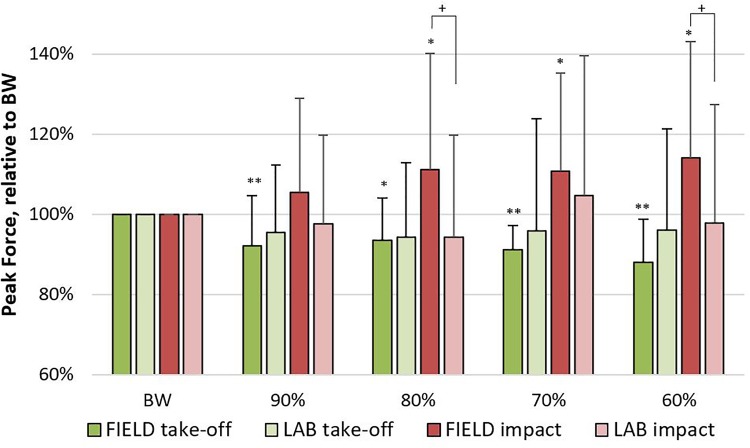
Data are expressed as mean ±*SD*. Peak countermovement jump take-off force and peak impact force with 90, 80, 70, and 60% of bodyweight (BW), expressed as a percent relative to BW jumps during field-(_FIELD_) and lab-based (_LAB_) testing. Moderate effect (*d* = *0.5–0.79*) compared to BW (^∗^), large effect (*d* ≥ 0.80) compared to BW (^∗∗^), and moderate effect between set-ups at the same bodyweight (^+^) (*d* = *0.5–0.79*).

**FIGURE 6:**
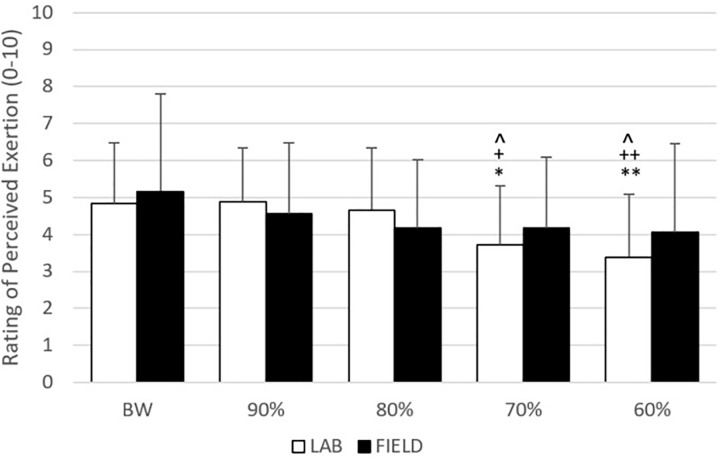
Rating of Perceived exertion (RPE) data are expressed as mean ± *SD*. Moderate (^∗^) and large (^∗∗^) effect compared to BW, moderate (*d* = *0.5–0.79*) (^+^) and large (*d* ≥ *0.80*) (^++^) effect compared to 90%, and moderate (*d* = *0.5–0.79*) (^∧^) effect compared to 80%. No moderate or large effects were present between set-ups at the same bodyweight.

The mean ±*SD* for CT, FT, TOF, and IF are presented in **Figures 4**, **5**. For CT and FT, there were main effects for assistance level within each set-up and between set-ups at the same assistance levels (**Figure 4**). For TOF and IF, there were no significant interactions or main effects. There were no effects for IF in _LAB_, but there were moderate effects for IF between set-ups at 60 and 80% and within _FIELD_ at 60, 70, and 80%. There were also moderate to large effects for TOF in _FIELD_, but no effects in _LAB_ (**Figure 5**). For RPE, there were no significant interactions or main effects. Moderate to large effect sizes were present for RPE during _LAB_ but were only negligible or small during _FIELD_ (**Figure 6**).

## Discussion

**FIGURE 7:**
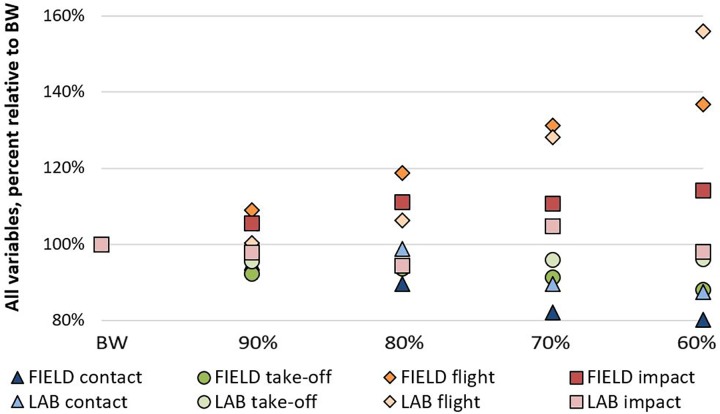
Mean values of ground contact time, peak take-off force, flight time, and peak impact force. Data are shown without standard deviations or statistical significance to clearly present changes in variables across bodyweight (BW) conditions.

Our results show that certain kinetic variables of assisted jumping were noticeably different (CT and FT) between _FIELD_ and _LAB_ compared to other variables where the differences are still likely present but perhaps less apparent (TOF and IF) (**Figure 7**). As coaches may choose to implement assisted jumping using commercially available resistance bands, this study highlights the importance of implementing an exercise to achieve a desired training stimulus and that field-based methods may not function the same as similar laboratory-based methods.

### Ground Contact Time

In our study, CT decreased at 80% with an additional but similar decrease at 70 and 60% in _FIELD_, but only decreased, and to a similar amount, at 70 and 60% in _LAB_. Additionally, CT was not different between BW and 90% for either _FIELD_ or _LAB_. Therefore, if the aim of assisted jumping is to be “quicker off the ground” as many coaches hypothesize and yearn for, bodyweight should likely be reduced to 80%, or possibly 70% depending on the set-up being used, as assisted jumping at 90% of bodyweight did not affect CT in either method. Previous research has shown that the ground contact time during an elite running vertical jump can be from 230 to 350 ms and elite running long jump can be from 150 to 170 ms (Stefanyshyn and Nigg, 1998), both of which are much quicker than the average CT of 696 ms during the BW countermovement jumps of the present study. At 60% BW, CT was reduced to an average of 571 ms in _FIELD_ and 600 ms _LAB_ but would still be considered as a “slow plyometric” exercise (i.e., ground contact > 250 ms). Therefore, although assisted jumping reduces CT compared to BW jumping, which has also been shown to occur after a period of assisted jump training (Makaruk et al., 2014), future research should investigate assisted jumping during other, faster plyometric exercises (i.e., ground contact < 250 ms) that place less of a focus on hip and knee extension, like countermovement jumps, and more of a focus on ankle plantar flexion such as ankle hops (Holcomb et al., 1996).

Regardless of the slow plyometric nature of countermovement jumps, the _FIELD_ condition of the present study resulted in less CT than _LAB_ at 80, 70, and 60%. Therefore, this interesting finding substantiates the need for the present study. It is likely that with the short bands in _FIELD_, the level of assistance rapidly increases as their length increases during the countermovement; on the other hand, with the _LAB_ set up, the level of assistance remains more, or less constant regardless of their stretch length. As a result, it is possible that more of the subjects’ inertial energy was absorbed during the countermovement in _FIELD_ than in _LAB_, allowing the subjects to have a shorter amortization phase during _FIELD_. Another possible explanation comes from a previous study implementing a long elastic band similar to _LAB_ (Markovic and Jaric, 2007) that showed that although ground contact time was not different between a bodyweight condition and 70% of bodyweight, the 70% condition resulted in greater eccentric depth during the countermovement (Markovic et al., 2007). Therefore, it is possible that the eccentric depth of the _LAB_ assisted counter-movements in the present study may have been deeper than in _FIELD_, resulting in a greater CT during _LAB_, albeit still reduced compared to BW. However, these hypotheses remain purely speculative, as these data were not analyzed in the present study but should be in future research. Nevertheless, if CT decreases, it is possible that TOF would decrease because there is less time to generate ground reaction forces.

### Take-Off Force

In line with the aforementioned logic, TOF was 7–12% less during _FIELD_ compared to BW jumping, which also agrees with data of previous research that utilized an assisted configuration very similar to _FIELD_ (Argus et al., 2011). In that study, it was found that when bodyweight was reduced by approximately 28%, peak take-off force was about 10.7% less than bodyweight jumps, which is in accordance with our 70% _FIELD_ findings of approximately a 9% decrease in TOF. The lack of effect of assistance on TOF during _LAB_ (an insignificant 4–5% decrease) is interesting, as it would be expected that as the amount of assistance changes, the ground reaction forces would also change as they did in _FIELD_. However, it may be that the change in CT during _LAB_ was not great enough to play a significant role in altering TOF, as may have been the case during _FIELD_.

Although this relationship between CT and TOF is fairly simple and comprehensible, the possible change in eccentric depth may have also played a role in TOF. If in fact the eccentric depth during _LAB_ was deeper than in _FIELD_, subjects may have experienced a greater range of motion in which they could generate force during _LAB_ which would also correspond to the greater CT observed during _LAB_. If that line of thinking is true, a greater range of motion may have stimulated the stretch-shortening cycle to a greater extent during _LAB_ than in _FIELD_, resulting in a more rapid concentric phase. This interaction has also been hypothesized by others (Markovic et al., 2007), and is supported by previous research that showed increased total impulse with greater eccentric depths without a change in peak force (Cormie et al., 2009). Although these explanations are logical, such data was not measured in the present study, so future studies should aim to determine if this hypothesis holds true. If so, it would appear that set-ups similar to _LAB_ may be more beneficial for increasing concentric rate of force development compared to _FIELD_. However, coaches should understand that this rationale is only hypothetical and must be investigated. Regardless of the possible mechanisms at play, a combination of a longer CT, a greater possibility to produce concentric TOF, or a combination of both would likely result in a subsequent increase in jump height.

### Flight Time

As this was not a performance-based study and jump height was not a primary outcome of the present study, the design and laboratory set-up did not include such measurements. Nevertheless, it can be assumed that as FT increases, jump height increases. As hypothesized, FT progressively and continuously increased at all bodyweights in _FIELD_, but only started to increase from 80% in _LAB_. Unique to FT, a particularly interesting pattern can be observed (**Figure 4**) in that FT_FIELD_ was greater than FT_LAB_ at 90 and 80% BW, FT was similar between _LAB_ and _FIELD_ at 70% BW, and then FT_LAB_ was greater than FT_FIELD_ at 60% BW. This phenomenon may be partially explained by the resting and stretch length of the bands used in each set-up.

Although TOF plays a significant role in concentric acceleration and velocity, the assistance force of the bands cannot be disregarded. As the resting length of the _FIELD_ bands were shorter, the total stretch length needed to achieve a desired level of assistance was much less than the amount of stretch needed during _LAB_ to achieve the same assistance level. However, during the same countermovement distance, the _FIELD_ bands were stretched to a greater percentage of their resting length, which would have provided more assistance at the bottom of the countermovement. As a result, it is possible that although TOF force was decreased in _FIELD_, take off velocity may have increased, resulting in a greater FT which was observed in a previous study using a system similar to _FIELD_ (Argus et al., 2011). Also considering the resting and stretched length of the bands, it is likely that the longer total stretch of the _LAB_ bands during 60% resulted in a transfer of elastic energy not only during the concentric phase but even during the entire flight phase. Therefore, there was a constant upward force during _LAB_ that was not present in _FIELD_, as the bands often became fully relaxed near the apex of the jumps during. This has practical implications because coaches should be aware of their own unique assisted set-ups, as a greater or lesser stretch at the same initial level of assistance may result in different jumping kinetics and kinematics. Similar to the logic of increasing CT and providing more time to generate TOF, the same could be said of an increased FT allowing gravity more time to accelerate the body downwards and possibly increase IF.

### Impact Force

With that in mind, bodyweight assistance had an effect on IF, increasing it by 10–14% during _FIELD_ and only with greater assistance levels, but the magnitude of the increases in IF were not proportional to the increases in FT. Interestingly, this phenomenon did not happen during _LAB_, as IF remained statistically unchanged across all assistance levels, which may be attributed to the constant tension of the bands during LAB explained above, not only resulting in a longer propulsive elastic force but also resulting in a longer “breaking” force when coming back down to the ground. Our data is in stark contrast to that of one study in particular that utilized a similar design as _FIELD_ (Argus et al., 2011). They found that impact forces during landing were 36% greater following a bodyweight jump compared to an assisted jump with 72% of bodyweight using a very similar set-up to the _FIELD_ set-up of the present study. However, our data not only showed that BW jumps did not result in greater IF than assisted jumps, but that the opposite occurred with 60, 70, and 80%_FIELD_ resulting in approximately 14, 11, and 11% greater IF than BW.

Like many of the other variables analyzed in this study, the 90% condition did not affect IF, indicating that reducing bodyweight by 10% likely does not affect countermovement jump kinetics or kinematics. However, the seemingly greater IF during _FIELD_ compared to _LAB_ at the 60 and 80% conditions are difficult to explain, especially since the same did not occur at 70%. One study utilizing a set-up similar to _LAB_ observed that eccentric force (i.e., IF in the present study) remained unchanged after 7 weeks of assisted jump training, which would agree with our study assuming that repeated exposure to a stimulus results in a similarly specific long-term adaptation (Markovic et al., 2007). Another study indicated that impact forces relative to the jumping bodyweight (i.e., reduced bodyweight due to bungee assistance) actually increased as assistance increased over 20% when using a system that was similar to _LAB_, but included bands that were closer to the length of _FIELD_ (Tran et al., 2011). However, considering the large standard deviations and lack of a consistent pattern for IF in the present study, we believe the lack of a statistically significant difference and only moderate effects sizes indicate that further research is needed to determine the effects of assisted jumping on IF, especially with different modes of assistance.

### Ratings of Perceived Exertion

In recent years, a steady flow of research has been investigating the relationships between how athletes perceive training and the presence of physiological, neuromuscular, and performance fatigue (Foster et al., 2001; Morishita et al., 2014; Haddad et al., 2017; Tufano et al., 2017). In many cases, an athlete’s RPE correlates so well to these different measures of fatigue, that some coaches and researchers go so far as to prescribe training based on how an athlete feels (Helms et al., 2018a,b), a decision that more traditional periodization-driven strength and conditioning coaches may find astounding. Nevertheless, the application of RPE during resistance training is becoming more commonplace, but to our knowledge, this is the first study to implement an RPE scale during assisted training. As expected, RPE decreased in the present study as the bodyweight of an athlete decreased, but only during the 70 and 60% BW conditions of _LAB_, with no changes in RPE during _FIELD_.

Previous researchers have suggested that assisted jumping may be less physically and physiologically demanding than bodyweight or overload training (Markovic et al., 2013), which would agree with our study, considering the relationship between RPE and physiological stress (Pierce et al., 1993; Tufano et al., 2017) and the lower RPE values at greater assistance levels during _LAB_. However, it is interesting that RPE did not change during _FIELD_. One possible explanation is that our subjects reported their RPE for the previous set of countermovement jumps as a whole, not specifically just the RPE for the legs. As the hands were not actively involved during _LAB_ and passively held the harness, it is likely that subjects did not focus on their grip and only thought about how they subjectively perceived the actual jumps when reporting RPE (i.e., jumping with a decreased bodyweight is easier). However, as the hands played a more active role in holding the bands to the harness during _FIELD_, subjects may have considered this when rating their RPE. Therefore, it is possible that it was not difficult to hold the bands to the harness during the 90 and 80% _FIELD_ conditions, whereas the 70 and 60% _FIELD_ conditions put a lot more stress on the hands to keep the bands secured to the harness. With that being the case, it is likely that the perceived increased effort of the hands may have canceled out any possible decrease in perceived effort of the legs, resulting in a more stagnant, systemic RPE. Future researchers should determine whether other methods of securing resistance bands to a subject alter the RPE, as these findings could be useful during training.

### Practical Implications and Study Limitations

This study highlights multiple important training considerations that, until now, have not been addressed within the strength and conditioning literature. First, it is important that coaches understand the effects of performing similar exercises using different modes and that they be cognizant when implementing specific exercises to achieve desired training outcomes. For example, if coaches aim to utilize assisted jumping to facilitate an overspeed stimulus and decrease ground contact time, it appears as though athletes should use enough assistance to decrease their bodyweight by 30 or 40% for the greatest effect, although previous studies have shown that as little as 10% (Imachi et al., 1997), or 10 kg (Sheppard et al., 2011) of assistance results in increases in jump height. However, our data also show that different band types and lengths will likely result in different ground contact and flight times in practice despite the initial assistance levels being equal, meaning that coaches may wish to choose specific bands and assistance levels for specific training stimuli. As such, forces during field-based assisted jumping, especially with shorter bands and greater levels of assistance, may not correspond to the values that can be found in the literature that are derived from other lab-based set-ups. Additionally, our study only utilized a field-based set-up whereby subjects grabbed a single resistance band with each hand, with the manufacturer stating that each band can provide up to 50 kg of resistance (or assistance in this case). Therefore, it is possible that the length, width, stiffness, resistance, and quantity of the bands used in practice could result in different kinetics and kinematics. As a result, coaches should strive to measure the kinetics of their own assisted methods to properly prescribe plyometric assisted training.

Additionally, although RPE may have been overshadowed by kinetic variables in this paper, it is important for coaches to realize that step-wise reductions in bodyweight are likely not perceived at the same rate as step-wise increases in external load. As previous studies have mentioned, RPE is a valid and reliable tool for monitoring, or even prescribing, training (Foster et al., 2001; Day et al., 2004). However, this does not seem to be the case with assisted jumping, possibly because the physiological and metabolic demands of assisted training may be lower than those of bodyweight or overload tasks (Markovic et al., 2013), which athletes are often used to and perform regularly. As both of these rationales were solely hypothetical and as this study is, to our knowledge, the first study to measure RPE during assisted training, future research should further investigate the use of RPE and other related scales during assisted training.

Lastly, this study did not determine the long-term effects of lab-based and field-based assisted jumping on ground contact time, and future studies should investigate whether these different assisted countermovement jump training set-ups translate into improved performance. Although this study did not implement a longitudinal design, it is important to translate these acute findings into practice to build on the current body of assisted training literature (Argus et al., 2011; Markovic et al., 2011; Sheppard et al., 2011).

## Conclusion

In conclusion, if the purpose of utilizing assisted jumping is to decrease ground contact time without altering jumping and landing forces, lab-based systems may be a better option, especially when using greater levels of assistance. However, if maintaining forces is not a priority, field-based systems are likely sufficient. Additionally, _LAB_ and _FIELD_ systems resulted in different CT, FT, and IF at different levels of assistance, highlighting the fact that methods used by coaches should be assessed in their own way in order to achieve the desired training stimuli.

## Data Availability Statement

The raw data supporting the conclusions of this manuscript will be made available by the authors, without undue reservation, to any qualified researcher.

## Author Contributions

All authors participated in all aspects of the present study: data collection, data analysis, and creating the manuscript.

## Conflict of Interest Statement

The authors declare that the research was conducted in the absence of any commercial or financial relationships that could be construed as a potential conflict of interest.
